# Endogenous Estrogen Influences Predator Odor-Induced Impairment of Cognitive and Social Behaviors in Aromatase Gene Deficiency Mice

**DOI:** 10.1155/2021/5346507

**Published:** 2021-09-20

**Authors:** Yaner Gao, Lei Ma, Feng Gao, Zuoli Sun, Zhengrong Zhang, Yuhong Li, Rena Li

**Affiliations:** ^1^Beijing Institute for Brain Disorders, Capital Medical University, Beijing, China; ^2^The National Clinical Research Center for Mental Disorders & Beijing key Laboratory of Mental Disorders, Beijing Anding Hospital, Capital Medical University, Beijing, China; ^3^School of Life Sciences, University of Science and Technology of China, Hefei, China

## Abstract

Epidemiological studies have suggested that traumatic stress increases vulnerability to various mental disorders, such as dementia and psychiatric disorders. While women are more vulnerable than men to depression and anxiety, it is unclear whether endogenous estrogens are responsible for the underlying sex-specific mechanisms. In this study, the aromatase gene heterozygous (Ar+/-) mice were used as an endogenous estrogen deficiency model and age- and sex-matched wild type mice (WT) as controls to study the predator odor 2,3,5-trimethyl-3-thiazoline- (TMT-) induced short- and long-term cognitive and social behavior impairments. In addition, the changes in brain regional neurotransmitters and their associations with TMT-induced changes in behaviors were further investigated in these animals. Our results showed TMT induced immediate fear response in both Ar+/- and WT mice regardless of sexes. TMT induced an acute impairment of novel object recognition memory and long-term social behavior impairment in WT mice, particularly in females, while Ar+/- mice showed impaired novel object recognition in both sexes and TMT-elevated social behaviors, particularly in males. TMT failed to induce changes in the prepulse inhibition (PPI) test in both groups. TMT resulted in a slight increase of DOPAC/DA ratio in the cortex and a significant elevation of this ratio in the striatum of WT mice. In addition, the ratio of HIAA/5-HT was significantly elevated in the cortex of TMT-treated WT mice, which was not found in TMT-treated Ar+/- mice. Taken together, our results indicate that TMT exposure can cause cognitive and social behavior impairments as well as change catecholamine metabolism in WT mice, and endogenous estrogen deficiency might desensitize the behavioral and neurochemical responses to TMT in Ar+/- mice.

## 1. Introduction

Epidemiological studies have indicated that dramatic stress in early life may increase the risk for some major psychiatric disorders such as anxiety, major depression, schizophrenia, and posttraumatic stress disorder [1–4]. In addition, clinical studies have suggested that psychiatric patients with a history of early life stress often have more severe psychotic symptoms as well as impaired brain functions [5, 6]. While clinical studies have revealed the difference in the incidence of several psychiatric disorders between males and females, the difference in stress-related behaviors between males and females and the underlying mechanism remain unclear.

Various animal models have been developed for the investigations of dramatic stress. Predator odor fears conditioning is one of the most common models for the investigation of dramatic stress in rodents [7, 8], while many other models are also employed to investigate the maternal separation [9], immobilization stress [10], restraint stress [11], and drug stimulation [12] in various studies. 2,3,5-Trimethyl-3-thiazoline (TMT), a sulfur-containing odor isolated from fox feces [13], is one of the most widely used predator odor stressor. Accumulating evidence suggests that TMT exposure is suitable to investigate the uncontrollable stress responses in rodents [14, 15], such as the activation of a distinct neural circuit in various brain regions in stress response [14], the activation of hypothalamus-pituitary-adrenal (HPA) axis [16, 17], and induction of reliable fearful responses as freezing, diminished grooming behaviors, and decreased exploratory behavior [18, 19]. Although it is known that early life predator odor exposure may affect later life behaviors [20], the long-term effect of early life exposure to predator odor on the neurobiological behavioral system are still poorly understood. In addition, the effects of predator stress on the anxiety behaviors are still controversial as a delayed attenuation of anxiety in juvenile mice [21] and an increased anxiety in adolescence rats [8].

Men and women tend to react differently to stress [22]. Women are twice as likely as men to suffer from stress-related mental illnesses such as depression [23] and posttraumatic stress disorder [24]. It is also known that the fear response is different between males and females [25], and a sex-specific behavioral response to stress has also been reported in rats [26–29]. Furthermore, animal studies have demonstrated that estrogen can amplify the response of prefrontal cortex to stress, thus making females more vulnerable to the detrimental consequences to the stress [30] However, the role of endogenous estrogen in fear behaviors and its impact on the stress-related regional biological changes in the brain are still unclear. In the present study, the effects of predator odor exposure on the behaviors as well as brain regional neurotransmitter changes, which are critical for onset of psychiatric disorders as well as for the stress-related changes in brain regions for cognition, reward, and emotion, were investigated in adolescent mice [31, 32]. The mice with aromatase gene knockout (heterozygous; Ar+/-) were used as a model of endogenous estrogen deficiency to study the endogenous estrogen dependency on the TMT-induced short- and long-term effects on the behaviors as well as the neurochemical changes in three brain regions (the cortex, hippocampus, and striatum).

## 2. Methods

### 2.1. Subjects

All experiments were conducted according to the Guideline for the Use and Care of Laboratory Animals of the University of Science and Technology of China and the National Institutes of Health Guide for the Care and Use of Laboratory Animals (National Research Council (US) Committee for the Update of the Guide for the Care and Use of Laboratory Animals, 2011).

The Ar-/- C57BL/6J mice were generated by deleting exons 1 and 2 of the Cyp19 gene as preciously described [33]. Heterozygous mice (Ar+/-) were generated by breeding homozygous null male mouse with female wild-type (WT) mouse. Age- and sex-matched WT mice were used as controls. All mice were maintained with a 12 h light-dark cycle at a constant temperature (24 ± 1°C) with the humidity at around 40%. Mice were housed in cages (4 mice per cage) and given ad libitum access to food and water. A total of 53 mice aging 4-8 weeks (WT, *n* = 31; Ar+/-, *n* = 22) were randomly divided into the TMT (Sigma-Aldrich) exposure group and control odor diethyl phthalate (DEP, Sigma-Aldrich) exposure group. The numbers of male and female mice were similar between groups.

### 2.2. Behavioral Test

Animals were allowed to accommodate to the environment for 1 week, during which each mouse was handled for approximately 1-3 min daily for 7 days. Mice were transferred into the behavioral test room at least 30 min before test. Before the TMT exposure, all mice were subjected to open field test to exclude mice with significant hyper- or hypoactivities at baseline. Behavioral tests were performed at 24 h, 1 month, and 4 months after the TMT exposure. On the day of testing, mice were transferred to the test room 30 min prior to test and all behavioral experiments were conducted during the dark cycle. For the acute behavioral changes, mice were subjected to open field test, novel object recognition test, Y-maze test, and elevated plus maze 24 h after TMT exposure. For the investigation of short-term behavioral changes, mice were examined 1 month after TMT exposure with open field test, novel object recognition test, three-chamber social test, Y-maze test, elevated plus maze test, and prepulse inhibition test. For the investigation of long-term response to the TMT exposure, mice were examined with open filed test and elevated plus maze at 4 months after TMT exposure. Each mouse was examined in all the different tests at all time points. The results of all the behavioral tests were recorded and analyzed with a motion tracking system (EthoVision XT. 8.5, Noldus Netherlands). At the end of behavioral experiments, mice were sacrificed, and brain tissues were harvested and stored at -80°C until assay.

#### 2.2.1. Predator Odor Exposure

Mice were exposed to TMT or DEP as described previously with minor modification [34]. In brief, mice were placed in a 35 × 15 cm rectangle plastic box covered with Plexiglas. Then, mice were exposed to 20 *μ*l of undiluted TMT on a 2 × 2 cm filter paper at one end of the box for 10 min and the fear-related behaviors were recorded by videotape. The control animals were tested in the same manner except with 20 *μ*l of undiluted DEP. Mice were returned to their cages after the odor exposure. The testing box was cleaned with 70% ethanol solution and dried between two tests. The experiment was conducted in a fume hood located in an isolated test room to avoid any interference.

#### 2.2.2. Open Field Test

The open field test was performed as described previously with modifications [35]. In brief, the mouse was gently placed at the center of an open field box (50 × 50 × 50 cm) under a dim light and allowed to move freely for 5 min. The total distance moved was recorded and analyzed. The floor of the open field box was cleaned with 70% ethanol solution and dried between two tests.

#### 2.2.3. Novel Object Recognition Test

The test was conducted as described previously with minor modification [36]. All mice were allowed to accommodate to the environment for 5 min in a plastic cage (50 × 50 cm) with no objects. Twenty-four hours later, mice were allowed to explore two identical objects placed along one side for a total of 10 min. Followed by test session 24 h later, mice were allowed to explore the arena with one familiar object and one novel object presented at the location for 5 min. After the test of each animal, the box and objects were cleaned with 70% ethanol solution and dried. Exploration was defined as sniffing the object within 2 cm or touching it. Data are also expressed as a discrimination index, calculated according to the following formulae: (time exploring novel object − time exploring familiar object)/(time exploring novel object + time exploring familiar object).

#### 2.2.4. Y-Maze Test

The Y-maze is a Y-shaped apparatus with three arms at a 120 angle from each other, and each arm is measured length × width × height as 30 × 8 × 17 cm. The experiment was conducted as previously reported [37]. The animal was placed at the center of the apparatus and then allowed to explore the three arms for 5 min. An entry was counted when all four paws of the mouse were within the arm and the animal's snout was oriented toward the end of the arm. A spontaneous alternation is defined as a mouse entering a different arm of the maze in each of 3 consecutive arm entries, calculated with the following formula: spontaneous alternations/(total number of arm entries − 2). The apparatus was cleaned with 70% ethanol solution between each test and dried.

#### 2.2.5. Elevated plus Maze

The apparatus consisted of two open arms (28 cm × 5 cm), two closed arms (28 cm × 5 cm), and a central platform (5 × 5 cm) and was 65 cm high from the floor. The experiment was conducted according to previously reported [38]. Mice were placed on the central platform facing one of the open arms and allowed to explore the maze for 5 min. The time and entries to the open arms were quantified.

#### 2.2.6. Three-Chamber Social Test

The experiment was carried out according to previously reported [39]. The test was performed in a rectangular box (length × width × height = 60 × 40 × 22 cm), separated by two Plexiglas dividing walls with an opening to the middle chamber, which allows free access to each chamber. The test consisted of three sessions: habituation session, social preference (or sociability) session, and social novelty (or social recognition) session. The first session began with 5 min habituation in the center chamber followed by the second session for 10 min in which the mouse could freely explore all three chambers. During social preference session, the mouse was then gently confined in the center chamber while two mesh-wire target cages were placed at both sides of the box (one cage is empty and the other contained a genotype and sex-matched stranger mouse, stranger 1). The mouse was then allowed to freely explore all three chambers for 5 min. At the social novelty session, the mouse was again confined in the center chamber while an unencountered genotype and sex-matched stranger mouse (stranger 2) were placed in the empty cage. The mouse was then allowed to freely explore all three chambers for 5 min. Time spent in each chamber was recorded.

#### 2.2.7. Prepulse Inhibition Test

The experiment was carried out according to previously reported with minor modification [40]. Animals were allowed to accommodate to the apparatus without white noise for 10 min per day for 3 days. On day 4, the experiment began with a 5 min habituation period of 65 dB white noise that served as the background noise during the test. The habituation period was followed by 30 pulse-alone trials. The pulse-alone stimulus was a 40 ms presentation of 105 dB white noise. The prepulse period consisted of 10 presentations of pulse-alone stimulus and 10 presentations of each of four types of prepulse trials, presented in a random order with an interval of 10-30 s. The prepulse trials consisted of a 20 ms white noise that was either 75 dB or 88 dB and preceded the startle pulse by either 30 or 100 ms. Each level of intensity and timing of the prepulse were combined to form four types of prepulse trials. The prepulse period was used to calculate %PPT. %PPI = [(mean peak amplitude on pulse alone sessions–mean peak amplitude on pre pulse sessions)/(mean peak amplitude on pulse alone sessions)] × 100.

#### 2.2.8. High-Performance Liquid Chromatography (HPLC) Assay

Mouse brain tissues were collected and homogenized in 0.2 ml ice-cold perchloric acid (0.4 M), followed by centrifugation at 12,000× g for 20 min, 4°C. The supernatants were filtered through 0.22 mm Cellulose filters (Millipore, USA). The resulting solution was injected into the HPLC system for electrochemical detection (Model 5600A; Coularray Detector System, ESA, Chelmsford, MA, United States) [41, 42]. The neurotransmitters analyzed included dopamine (DA), 3,4-dihydroxyphenylacetic acid (DOPAC), 5-hydroxytryptamine (5-HT), 5-hydroxyindoleacetic acid (5-HIAA), and homovanillic acid (HVA) and were expressed as *μ*g/g protein. DA and 5-HT turnover rates were calculated as DOPAC/DA and 5-HIAA/5-HT ratios, respectively.

### 2.3. Statistical Analysis

All data are expressed as mean ± standard error (SEM). The behavioral data between the TMT group and DEP group in each genotype were compared by using 2 × 2 analysis of variance (ANOVA) with the alpha level at 0.05. Post hoc analysis was done using Bonferroni's test. The molecular data between groups in each genotype were compared by using the two-tailed *t*-test with the alpha level at 0.05. All the data were plotted using Prism software.

## 3. Results

### 3.1. TMT-Induced Immediate Fear Response and Acute Anxiety Impairment in Both WT and Ar+/- Mice

**Figure 1:**
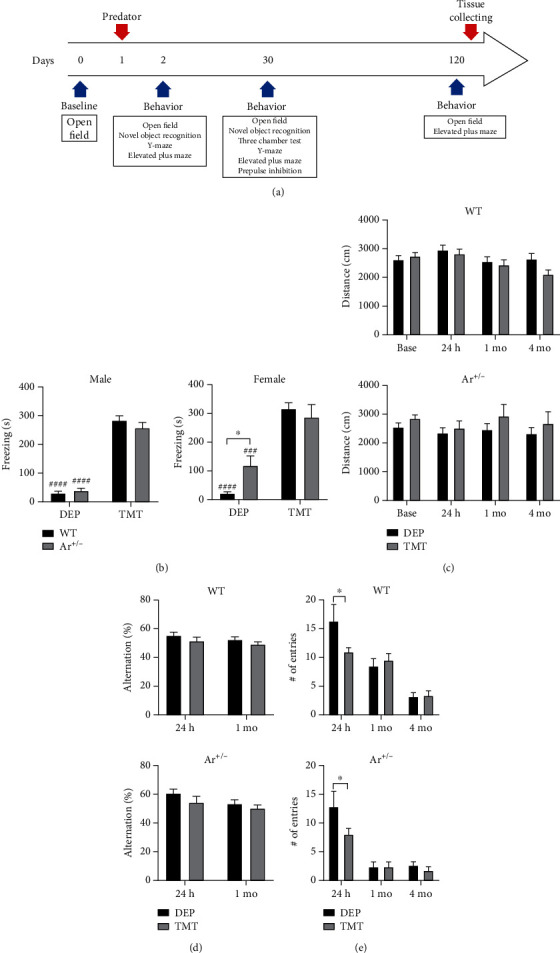
Procedures of experiments and behavioral changes after TMT exposure. (a) Procedures of experiments. (b) Freezing time increased significantly after TMT exposure in two genotypes in both sexes. (c) Open field test and (d) Y-maze test at baseline, 24 h, 1 month, and 4 months after TMT exposure in the WT mice and Ar+/- mice. (e) Results of plus maze test at 24 h, 1 month, and 4 months after TMT exposure in both WT mice and Ar+/- mice. Data are shown as mean ± SEM; ^####^*P* < 0.0001, ^###^*P* = 0.0008 vs. TMT-treated mice. ^∗^*P* < 0.05 between groups as marked.

To investigate whether endogenous estrogen alters the freezing levels in response to a single TMT exposure, the freezing time was recorded as previously described [43]. The experimental condition and design were described in Figure 1(a). Results showed the freezing levels were significantly higher in both WT and Ar+/- mice, and there was no significant difference in the TMT-induced freezing time between WT mice and Ar+/- mice regardless sexes (WT male mice: *P* < 0.0001; Ar+/- male mice: *P* < 0.0001. WT female mice: *P* < 0.0001, Ar+/- female mice: *P* = 0.0008). In addition, a significant reduction was observed in the number of entry in the elevated plus maze test 24 h after TMT exposure in both WT mice (*P* = 0.0336) and Ar+/- mice (*P* = 0.0485) (Figure 1(e)). TMT exposure failed to induce acute, short-term and long-term changes of behaviors in the open field test and Y-maze test after TMT exposure in both WT mice and Ar+/- mice (Figures 1(c) and 1(d)).

### 3.2. TMT-Induced Genotype-Specific Impairment of Recognition Memory

**Figure 2:**
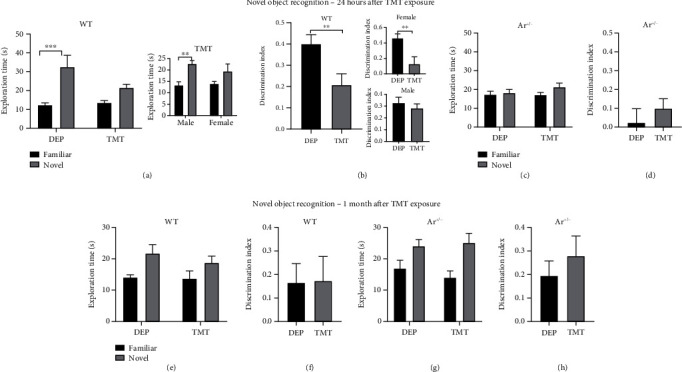
TMT-induced acute and chronic changes in the novel object recognition test in WT mice and Ar+/- mice. WT mice showed shorter time spent in exploring the novel object (a) and lower discrimination index (b) 24 h after TMT exposure, particularly in females, while the Ar+/- mice showed genotype-specific impairment of preference to novel object 24 h after TMT exposure (c, d). One month after TMT exposure, there were no significant differences between the DEP group and TMT group in the exploration time and discrimination index in the WT mice (e, f) and Ar+/- mice (g, h), respectively. Data are shown as mean ± SEM. ^∗^*P* < 0.05, ^∗∗^*P* < 0.01, and ^∗∗∗^*P* < 0.001.

The acute and short-term effects of stress on the memory were evaluated by the novel object recognition test 24 h and one month after TMT exposure, respectively. Two-way ANOVA revealed that WT mice showed significantly impaired memory 24 h after TMT exposure (DEP: *P* = 0.0002, TMT: *P* = 0.2355, Figure 2(a)). Moreover, the TMT-induced impaired recognition memory in WT mice was mainly found in females (male: *P* = 0.0051, female: *P* = 0.1602; Figure 2(a)). This indicated that only female WT mice lost interest in exploring a novel object as compared to male mice which remained a significant preference for exploring the novel object. TMT exposure significantly reduced the discrimination index (DI) which was calculated by the time spent on the novel object minus the time spent on the familiar object, followed by division by the total object exploration time in WT mice (*P* = 0.0083, Figure 2(b)). Similarly, the impairment of recognition behaviors was sex-specific because it was only noted in female WT mice (female: *P* = 0.0093, male: *P* = 0.4949; Figure 2(b)). In contrast, Ar+/- mice showed genotype-specific impairment of recognition memory (Figure 2(c)). Ar+/- mice had no interest in exploring a novel object regardless of stimulation (TMT or DEP) evidenced by the absence of significant differences in the exploration time and DI. To investigate the short-term effect of TMT on the recognition behaviors, the mice were tested one month after TMT exposure. Interestingly, the effect of TMT on the novel object recognition memory disappeared one month after TMT exposure in the WT mice (Figures 2(e) and 2(f)), while Ar+/- mice maintained the similar failure in any preference for the novel object 24 h and 1 month after TMT exposure (Figures 2(g) and 2(h)).

### 3.3. Genotype-Specific Effects of TMT on Social Behaviors

**Figure 3:**
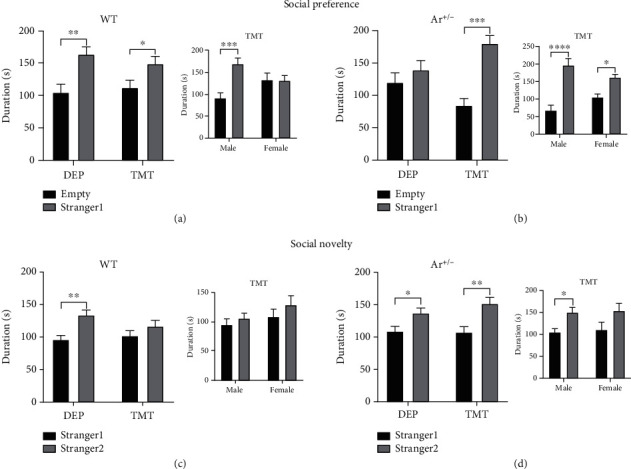
TMT-induced social preference and social novelty in the three-chamber social test. TMT exposure compromised the social preference (a) in the WT mice, particularly in females, as well as the social novelty (c). The Ar+/- mice after TMT exposure promoted the sociability (b) and social novelty (d), particularly in males. Data are expressed as mean ± SEM. ^∗^*P* < 0.05, ^∗∗^*P* < 0.01, and ^∗∗∗^*P* < 0.001.

To investigate the effect of endogenous estrogen on the stress-induced changes of social behaviors, WT mice and Ar+/- mice were subjected to 3-chamber social interaction test 1 month after TMT exposure. As rodents normally prefer to spend more time with another rodent rather than with an inanimate object as social preference and intend to explore a novel intruder more than a familiar one as social novelty [44], both social preference and social novelty were examined in the WT mice and Ar+/- mice. Results showed the TMT-induced changes in social behaviors were genotype-specific. Specifically, WT mice displayed normal preference toward stranger mice as compared to an empty chamber in both the control-treated mice and TMT-treated mice (control-treated mice: *P* = 0.0017, TMT-treated mice: *P* = 0.0454; Figure 3(a)). Furthermore, TMT-exposed WT mice showed impairment of social preference, only in the females, but not in the males (males: *P* = 0.001, females: *P* > 0.9999; Figure 3(a)). Interestingly, a different response to the TMT exposure was observed in the Ar+/- mice. As shown in Figure 3(b), the Ar+/- mice failed to show interest in the stranger mouse in the DEP group but an enhanced social preference was observed in the TMT group (control-treated mice: *P* = 0.6150, TMT-treated mice: *P* < 0.0001), and especially, the male Ar+/- mice showed significantly increased time spent with the stranger mice (males: *P* < 0.0001, females: *P* = 0.0388; Figure 3(b)). Similar results were found in the social novelty test. WT mice in the DEP group showed significantly increased time in the chamber spent with a new mouse (stranger 2) over the chamber containing a familiar mouse (stranger 1) (*P* = 0.0029, Figure 3(c)). The TMT exposure caused the impairment of social novelty in the WT mice (*P* = 0.3727, Figure 3(c)) in both males and females (Figure 3(c)). In contrast, TMT exposure failed to change the social novelty in the Ar+/- mice as both stressed and control Ar+/- mice spent more time with the novel mice (stranger 2) relative to the familiar mice (stranger 1) (DEP-treated mice: *P* = 0.0456, TMT-treated mice: *P* = 0.0011; Figure 3(d)). However, TMT exposure impaired the social novelty in the female Ar+/- mice, but not in the male ones (male mice: *P* = 0.0472, female mice: *P* = 0.0877; Figure 3(d)).

### 3.4. Effect of TMT Odor Stress on PPI in WT and Ar+/- Mice

**Figure 4:**
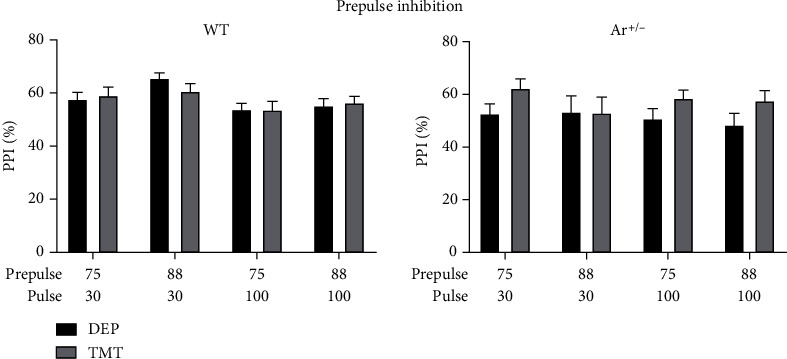
TMT exposure had no influence on the PPI in WT mice and Ar+/- mice. Animals were tested with various white noise level/interval time combinations (75 dB/30 ms, 88 dB/30 ms, 75 dB/100 ms, and 88 dB/100 ms).

PPI is an operational measure for sensorimotor gating, which is a known deficit in schizophrenia [45]. To investigate whether endogenous estrogen deficiency alters the stress-related sensorimotor gating, the PPI was examined between TMT-treated and DEP-treated WT and Ar+/- mice. Four types of prepulse trials were tested. As shown in Figure 4, there were no significant effects of TMT exposure on the PPI between WT mice and Ar+/- mice regardless of sexes.

### 3.5. Effects of TMT on the Catecholamine Metabolism

Mice were sacrificed by decapitation four months after TMT exposure. Catecholamine metabolites were examined in various brain regions by HPLC. Our results show that TMT exposure failed to alter the brain levels of DA, DOPAC, HVA, 5-HT, and HIAA in different brain regions (i.e., the cortex, striatum, and hippocampus) in both genotypes (Tables 1 and 2). However, when the 5-HT and DA turnover was analyzed, TMT exposure resulted in a slight increase of the DOPAC/DA ratio in the cortex (Table 1, two-tailed *t*-test, *P* = 0.0539) and a significant elevation of the ratio in the striatum (Table 1, two-tailed *t*-test, *P* = 0.0362), respectively, in the WT mice. In contrast, the ratio of HIAA/5-HT was elevated only in the cortex (Table 1, two-tailed *t*-test, *P* = 0.0464). In the Ar+/- mice, TMT exposure induced a light elevation of the DOPAC/DA ratio only in the hippocampus (Table 2, two-tailed *t*-test, *P* = 0.0511), while 5-HIAA/5-HT ratio remained unchanged regardless brain regions (Table 2).

## 4. Discussion

Accumulating evidence suggests that psychosocial stress is associated with an increased risk for psychosis. For example, people are more likely to develop psychosis if they have decreased tolerance to stress [46]. In addition, stress can precipitate the onset or relapse of psychosis [47, 48]. Since females have higher prevalence of depression and anxiety, the present study investigated the effects of endogenous estrogen on the stress-induced changes of behaviors (including cognition, anxiety, social interactions, and psychosis-related PPI).

First, results showed TMT induced similar fearful responses in both WT mice and Ar+/- mice (Figure 1(b)), suggesting 10 min TMT exposure causes a traumatic stress to mice and low estrogen level appears to have little influence on the stress-induced fear response. In addition, there were no differences in various behavioral responses to TMT exposure between WT mice and Ar+/- mice (Figures 1(c)–1(e)) at different time points. We speculate that ceiling effects occur in the present study. As shown in Figures 1(c)–1(e), stress has minimal impact on these behaviors.

In contrast, the novel object recognition test showed TMT exposure induced a sex-specific change in the behaviors of WT mice, such as more exploration time on the novel object than on the familiar object in males and lower DI in female WT mice (Figures 2(a) and 2(b)), while no effect of TMT was observed on the novel object recognition behaviors in Ar+/- mice 24 h after TMT exposure (Figures 2(c) and 2(d)). This sex-specific difference in the TMT-induced behavior change in WT mice was noted only 24 h after TMT exposure and disappeared 1 month after TMT exposure (Figures 2(e) and 2(f)). One month after TMT exposure, the Ar+/- mice remained unresponsive to TMT (Figures 2(g) and 2(h)). The TMT-induced acute novel object recognition change seems to be unrelated to the overall exploration duration or anxiety-like behaviors since our results showed no difference in the exploratory behavior indexed by locomotion in the open field test and the number of entry or time spent in the EPM test, respectively, between TMT-treated mice and DEP-treated mice (Figures 1(c) and 1(e)). Thus, our results indicate that predator odor can disrupt working memory in the WT mice, which is consistent with previous findings from rats [49]. However, our study for the first time reports a change in the TMT-induced working memory deficit in animals with endogenous estrogen deficiency in vivo.

The three-chambered social test is widely used to examine the sociability and social recognition in rodents [50, 51]. Specifically, our results showed TMT exposure impaired the preference for social novelty (social recognition) without significantly impairing the investigation of an initially novel conspecific (sociability) in WT mice. We speculate that this selective impairment correlates with inherent differences between social recognition and sociability behaviors. Previous studies have demonstrated that exploration of a novel environment with no choice may promote sensation-seeking behaviors [52], whereas free choice to explore a familiar or novel environment promotes novelty-seeking behaviors [53]. Investigating one conspecific would represent sensation-seeking behaviors, because free choice between two different, discrete stimuli was eliminated. On the other hand, the investigation of familiar or novel conspecifics would represent a novelty-seeking behavior, because free choice was provided. It is important to note that novelty-seeking behavior under free choice conditions requires recall and recognition of familiarity versus novelty. Therefore, our results suggest that TMT exposure may selectively attenuate the social novelty-seeking behavior, which requires recognition memory, while sparing sensation-seeking behavior, which does not require recognition memory, in WT mice. This is supported by previous findings [54], and the novel object recognition test in the present study also showed stress impaired object recognition memory (novelty-seeking behavior) without obviously affecting total exploration time (sensation-seeking behavior) in the WT mice. Taken together, our results indicate that brief exposure to predator odor during adolescence impairs the social recognition of the WT mice. However, interestingly, TMT exposure failed to trigger similar behavioral changes in the Ar+/- mice. As shown in Figure 3(b), instead of reduced time spent with stranger 1 as in WT mice, the Ar+/- mice showed an enhanced sociability evidenced by more time spent with the stranger 1 than with the empty case as compared to the control Ar+/- mice. In social recognition test, the TMT-exposed Ar+/- mice also spent more time in the chamber with the novel stimulus mouse (stranger 2) than with a familiar stimulus mouse (stranger 1), indicating an improved social recognition behavior. In addition, the significantly elevated sociability and social recognition in Ar+/- mice were only observed in males, not in females (Figures 3(b) and 3(d)). To date, no studies have reported the influence of stress on the sociability and social recognition in the Ar+/- mice, and our study for the first time reported male-specific change in the social behaviors as a response to TMT exposure in the Ar+/- mice. It is known that aromatase is a key enzyme for the endogenous estrogen synthesis, and a significant reduction of estrogen level in female Ar+/- mice may increase the vulnerability to many diseases [55]. However, the reported changes of behaviors and outcome are conflicting in the male Ar+/- mice, such as improved cognition function and protection against Alzheimer's disease pathology due to the elevated endogenous testosterone level [56, 57]. In addition, recent animal studies show that estrogen amplifies stress-induced prefrontal cortex dysfunction in females, and such stress profiles include pharmacological stress [12] and restraint stress [30]. These findings suggest that estrogen may interact with stress-induced neurological dysfunction in females. The neurobiological basis for this enhanced sensitivity to stress in female mice might be related to a mixture effect of estrogen and testosterone, but the specific mechanism should be further investigated.

As PPI is an operational measure for sensorimotor gating which is known to be impaired in the schizophrenia patients, whether TMT exposure increases the risk for schizophrenia-like behaviors was investigated in the present study, and the PPI was examined one month after TMT exposure. As shown in Figure 4, predator odor failed to significantly alter the PPI response in the WT mice and Ar+/- mice (Figure 4), regardless of the different combinations of prepulse and pulse conditions. While the effects of stress on the PPI have been relatively understudied, previous studies have suggested a time window for the response to stress. For example, PPI is disrupted 24 h after natural predator (ferret) exposure, but not 48 h and 9 days after exposure [58]. In the present study, the time interval between TMT exposure and PPI test was one month, which might be outside of the time window for the response. However, whether other factors (such as the intensity and type of stress) also contribute to the less PPI response to TMT exposure in our study should be further investigated.

In addition, we also examined the brain levels and turnover of DA and 5-HT in various regions of WT mice and Ar+/- mice. Our results showed that TMT exposure slightly increased the DOPAC/DA ratio in the cortex and significantly elevated the ratio in the striatum of WT mice. In contrast, the DA level and DOPAC/DA ratio remained unchanged in different brain regions after TMT exposure in the Ar+/- mice (Table 1). This is consistent with previous findings that stress induces prefrontal cortex dysfunction by altering catecholamine release in male animals, particularly activating dopamine D1 receptor or norepinephrine *α*-1 receptor [59]. Likewise, other studies also reveal that psychological stress significantly alters the monoamine level in different brain regions, such as the prefrontal cortex, hippocampus, and amygdala [60, 61]. Similarly, our study showed a higher HIAA/5-HT ratio in the cortex after TMT exposure in the WT mice, while only elevated HIAA level was observed in the cortex of Ar+/-mice (Table 2). The DA and 5-HT metabolite levels remained unchanged in the hippocampus of both WT and Ar+/- mice, which may explain the less long-term effect of TMT exposure on the cognitive behaviors in our study. These results imply that estrogen may contribute to the sex difference in the behavioral performance in the presence of stress by interacting with neurotransmission pathways in the central nervous system (CNS), but the exact mechanism should be further elucidated. Taken together, our results along with previous findings suggest that estrogen may promote stress-induced behavioral and neurochemical responses, and reduced endogenous estrogen may desensitize the response to stress.

There were still limitations in the present study. Although our results indicated that endogenous estrogen deficiency might desensitize the behavioral and neurochemical responses to predator odor in the Ar+/- mice, the estrous stage was not determined in female animals in the present study. We speculate that the change in estrogen level due to aromatase deficiency far exceed the alteration of estrogen level in different estrus stages.

The effects of aromatase deficiency on the pubertal development of the reproductive system are still unclear. Animal studies have indicated that female ArKO mice aged 9 weeks display underdeveloped external genitalia and uteri [62]. Due to the low fertility in homozygous Cyp19 gene knockout mice, we did not include the ArKO mice as well as WT littermates in this study. It is assumed that aromatase deficiency would impair brain development which might explain a decreased sensitivity to the predator odor exposure in the Ar+/- mice. Further investigation is needed.

## 5. Conclusion

Brief predator odor exposure can induce short-term changes in the cognitive behaviors and long-lasting changes in the social behaviors in adolescent WT mice. The same stress also induces long-lasting changes of DA and 5-HT metabolism in the brain of WT mice. Interestingly, similar results are not observed in the Ar+/- mice. Our data suggest that the changes of DA and 5-HT neurotransmission in the brain may be related to behavioral changes secondary to a brief predator odor exposure, and reduced endogenous estrogen may desensitize the responses to stress-related behaviors and improve the changes in the brain neurotransmission.

## Figures and Tables

**Table 1:** The effects of TMT on DA and 5-HT system in brain regions of WT mice. Each value indicates the mean ± S.E.M of 9-13 mice.

Brain regions	DA (ng/g)		DOPAC (ng/g)		HVA (ng/g)		DOPAC/DA		5-HT (ng/g)		HIAA (ng/g)		HIAA/5-HT	
	DEP	TMT	DEP	TMT	DEP	TMT	DEP	TMT	DEP	TMT	DEP	TMT	DEP	TMT
Cortex	1492.11 ± 343.25	944.93 ± 214.28	735.74 ± 140.31	813.16 ± 126.46	252.84 ± 32.11	268.55 ± 43.04	0.69 ± 0.11	1.58 ± 0.39	417.57 ± 48.03	359.05 ± 42.09	411.11 ± 30.86	487.70 ± 22.90	1.07 ± 0.10	1.61 ± 0.22^∗^
Striatum	3991.64 ± 526.95	4164.83 ± 707.43	765.82 ± 84.48	859.70 ± 110.39	389.80 ± 38.71	429.86 ± 40.43	0.18 ± 0.00	0.23 ± 0.02∗	551.37 ± 45.85	533.97 ± 38.50	539.97 ± 56.29	586.70 ± 60.68	1.00 ± 0.10	1.13 ± 0.13
Hippocampus	54.71 ± 7.65	52.38 ± 6.02	73.38 ± 15.29	77.57 ± 7.61	—	—	1.50 ± 0.35	1.67 ± 0.25	436.24 ± 40.78	509.73 ± 68.44	558.32 ± 25.86	493.39 ± 51.93	1.45 ± 0.20	1.21 ± 0.11

-: not measured, ^∗^*p* < 0.05.

**Table 2:** The effects of TMT on DA and 5-HT system in brain regions of Ar+/- mice. Each value indicates the mean ± S.E.M of 4-8 mice.

Brain regions	DA (ng/g)		DOPAC (ng/g)		HVA (ng/g)		DOPAC/DA		5-HT (ng/g)		HIAA (ng/g)		HIAA/5-HT	
	DEP	TMT	DEP	TMT	DEP	TMT	DEP	TMT	DEP	TMT	DEP	TMT	DEP	TMT
Cortex	518.98 ± 142.57	819.44 ± 282.64	610.68 ± 233.59	1166.62 ± 293.49	241.85 ± 51.36	288.62 ± 64.94	1.04 ± 0.11	1.77 ± 0.48	295.53 ± 27.60	358.61 ± 49.10	433.88 ± 46.29	592.21 ± 48.80^∗^	1.51 ± 0.13	1.76 ± 0.16
Striatum	4165.55 ± 812.10	4756.18 ± 846.31	1042.69 ± 111.62	1117.03 ± 231.72	457.49 ± 32.28	445.94 ± 60.55	0.19 ± 0.01	0.24 ± 0.022	569.16 ± 47.92	575.46 ± 51.83	599.65 ± 67.01	758.65 ± 65.29	1.07 ± 0.10	1.33 ± 0.09
Hippocampus	74.45 ± 15.61	52.83 ± 11.38	94.62 ± 16.02	109.97 ± 15.94	—	—	1.60 ± 0.19	2.48 ± 0.28	471.08 ± 28.80	468.34 ± 17.38	532.93 ± 44.14	614.85 ± 23.52	1.17 ± 0.12	1.32 ± 0.07

-: not measured, ^∗^*p* < 0.05.

## Data Availability

The data used to support the findings of this study are available from the first author upon request.
